# Prickly Poppies Can Get Pricklier: Ontogenetic Patterns in the Induction of Physical Defense Traits

**DOI:** 10.1371/journal.pone.0096796

**Published:** 2014-05-06

**Authors:** Ryan P. Hoan, Rhys A. Ormond, Kasey E. Barton

**Affiliations:** 1 Department of Botany, University of Hawai’i at Mānoa, Honolulu, Hawai’i, United States of America; 2 Biology Department, Willamette University, Salem, Oregon, United States of America; College of Charleston, United States of America

## Abstract

Plant ontogeny is a common source of variation in defense and herbivory. Yet, few studies have investigated how the induction of physical defense traits changes across plant ontogeny. Physical defense traits are costly to produce, and thus, it was predicted that induction as a cost-saving strategy would be particularly favorable for seedlings, leading to ontogenetic declines in the inducibility of these traits. We tested for induction of three different physical defense traits (prickles, latex and leaf toughness) in response to mechanical defoliation and jasmonic acid application using prickly poppies (*Argemone glauca* and *A. mexicana*, Papaveraceae) as a model system. Genetic variation in the induction of physical defenses was tested using maternal sib-ships sampled from multiple populations. Both species induced higher densities of laminar prickles, although the magnitude of induction was much higher in the endemic Hawaiian prickly poppy, *A. glauca*, than in the cosmopolitan *A. mexicana*. The magnitude of prickle induction was also higher in young compared to older juvenile plant stages in *A. glauca*, demonstrating a strong role of ontogeny. Neither latex exudation nor leaf toughness was induced in either species. Although significant genetic variation was detected within and among populations for constitutive expression of physical defense traits in *Argemone*, there was no evidence for genetic variation in the induction of these traits. This study provides the first evidence for the induction of physical defenses in prickly poppies, emphasizing how an ontogenetically explicit framework can reveal new insights into plant defense. Moreover, this study illustrates how sister species comparisons between island vs. continental plants can provide new insights into plant functional and evolutionary ecology, highlighting a fruitful area for future research on more species pairs.

## Introduction

Plant ontogeny has been shown to play a fundamental role in the expression of defense traits and patterns of herbivory [1], [2], [3]. Ontogenetic patterns in plant defense traits occur in response to shifts in herbivore selection pressures over the lifetimes of plants [4], and as a result of developmental constraints and changes in resource allocation priorities [5]. Considerable attention has recently been paid to the characterization of ontogenetic trajectories of plant secondary chemicals [6] and tolerance to herbivory [7], but less is known about ontogenetic patterns in induced resistance.

Induced responses to herbivory are widespread and common having been documented in hundreds of plant species [8], [9]. Like constitutive defenses, induced defenses also demonstrate ontogenetic variation. In herbs, juvenile plants are generally more inducible than older plants [1], likely due to the inherently greater plasticity of seedlings [10]. In woody plants, there is evidence for the opposite pattern, that the inducibility of secondary chemicals increases with plant age [11]. Although there is considerable evidence that physical defense traits, such as spinescence, leaf latex, and sclerophylly, are induced by herbivores [12], [13], [14], [15], [16], we have a poor understanding of how the induction of physical defenses may shift across plant ontogeny [17].

Physical defenses are costly to express because of the large investment in structural carbohydrates needed to construct them [15], [18], [19]. Thus, plants should benefit from having these traits be inducible in order to reduce construction costs until needed. On the other hand, because the induction of physical defenses requires the development of new tissues with higher densities of the physical defense traits (e.g. leaves with higher densities of prickles), there is an inherent time lag to physical defense induction [20], [21], which may reduce its effectiveness at deterring herbivores in ecological time. To our knowledge, only two studies have examined the induction of physical defense traits within an ontogenetic context, and both showed that the induction of trichomes in *Mimulus guttatus* (Phrymaceae) increased with ontogeny [16], [22].

We investigated ontogenetic patterns in the induction of physical defense traits using the endemic Hawaiian prickly poppy, *pua kala*, (*Argemone glauca* Nutt. Ex Prain Pope) as a model system. Like other prickly poppies of the genus *Argemone* (Papaveraceae), *A. glauca* displays several different physical defense traits including prickles, latex and very tough, glaucous leaves. Previous research has revealed that leaf prickle densities, latex and tolerance of damage are highly variable among populations on different Hawaiian Islands, and that these traits show strong ontogenetic patterns in their constitutive expression [23]. Here we ask whether prickles, latex and leaf toughness, assayed as specific leaf area [24], are inducible, and whether their induction varies across early plant ontogeny, providing the first test of ontogenetic patterns in the induction of a suite of physical defense traits.

A secondary goal of the study was to test for the inducibility of defense traits as evidence that island endemics can be well-defended against herbivores. Due to the absence of some guilds of herbivores from insular islands, such as mammals and herbivorous reptiles and mollusks in Hawai’i, it has been hypothesized that defenses have become relaxed in island plants due to low herbivore selection pressure [25], [26], [27]. While there is some evidence for lower defenses in island vs. continental plants [28], [29], [30], this idea remains largely untested. Because induced responses require highly specialized and sometimes costly signaling pathways and trait expression [31], [32], [33], the presence of induction would be particularly strong evidence for intact defense strategies in island plants. Yet, no previous study has tested for induction in island endemics. We compared the inducibility of prickles, latex, and leaf toughness in *A. glauca* and its continental sister species, *A. mexicana* [34], as a first test of induced responses in island vs. continental plants. Although this represents a sample size of 1 (1 island vs. 1 continental species), it is an important first step to understanding how plant defense evolves on islands.

## Materials and Methods

### Ethics Statement

Neither *A. glauca* nor *A. mexicana* are threatened or endangered taxa, and so seed collection on public roadsides is not restricted. Collection of seeds on private land was granted by Lisa Raymond, curator at the Maui Nui Botanical Garden; and George Enuton, Park Ranger at the Pu’ukoholā Heiau National Historic Site.

### Study System

*Argemone* consists of 30–32 species of annuals and short-lived perennials native to dry, warm habitats in the Americas, with one species endemic to Hawai’i, *A. glauca* [34]. The Mexican poppy, *A. mexicana*, is native to Mexico, but is now a cosmopolitan weed, and has been found in small populations in disturbed sites in Hawai’i since at least 1958 [35]. Hybrids between *A. glauca* and *A. mexicana* have been reported but are rare [36]. Although research is scant, insects, particularly beetles (Coleoptera) appear to be the primary herbivores of prickly poppies in continental *Argemone* species [37], [38], but historical populations of *A. glauca* may have also experienced herbivory by the now extinct flightless ducks which were folivorous [39] and hypothesized to have been the selective drivers for the de novo evolution of prickles in the Hawaiian lobeliads [40]. Thus, although the Hawaiian populations of *A. glauca* and *A. mexicana* sampled for this study are currently interacting with the same assemblage of herbivores, the recent arrival of *A. mexicana* and the extinction of most native Hawaiian herbivores give the species very different evolutionary histories which we predict to have led to persistent differences in their defenses. Previous research on *Argemone* has mainly focused on chemical defense associated with alkaloids in the latex [41], [42], [43], [44], although none of this research includes *A. glauca*. Recent evidence that laminar prickles are induced by high light in *A. glauca* [45] suggests that they may also function in mitigating high light stress or maintaining water balance.

### Sampling Summary

Although not always examined, there is ample evidence that induction is genetically variable [22], [46], [47]. We tested for genetic variation in the induction of physical defense traits using the maternal sib-ship approach [48]. Seeds of *A. glauca* and *A. mexicana* were collected from the islands of Maui (2010) and Hawai’i (2011). Neither species is threatened or endangered, and seed collecting at these sites was not regulated. On Maui, *A. glauca* was collected from the vicinity of the Maui Nui Botanical Garden (20.89305, −156.48573), where an established and naturally regenerating population receives no particular cultivation or care, and *A. mexicana* was collected from a naturalized population along Pulehu Road in Kula (20.84566, −156.41082). On Hawai’i, *A. glauca* was collected from 3 sites: along two roadsides (19.41154, −156.01812; 19.84889, −155.93040) and at the Pu’ukoholā Heiau National Historic Site (20.025996, −155.820104). *Argemone mexicana* was collected on Hawai’i from a single roadside population (19.41154, −156.01810). To be conservative since the genetic structure is not known for these species, we consider each island to be a single population. Thus, both species were sampled from 2 populations (Maui and Hawai’i). On both islands, seeds were collected from plants at least 2 m apart and often more than 10 m apart. Seeds from a single maternal plant were stored separately, which constitutes a maternal sibship or “genetic family”.

### Experimental Design

Seeds were soaked in tap water for 36 hours to facilitate germination [49] and germinated in flats filled with equal parts Promix BX (67–75% Canadian sphagnum peat moss, perlite, dolomitic and calcitic limestone, macro- and micronutrients, *Glomus intraradices mycorrhizae inoculum*) and black cinder. Following germination, seedlings with at least one true leaf were transplanted into 1-gallon (4.4 L) pots filled with equal parts Promix BX and black cinder, supplemented with a single application of slow-release fertilizer (Osmocote).

Experiments were conducted in an open-air grow area attached to the St John Plant Sciences Building on the UH-Manoa campus. Plants were exposed to full sun and precipitation, but were provided with supplementary water daily. Plant location was re-randomized every week in order to minimize the effects of micro-environmental variation such as wind exposure. Because of variation in the germination rates of *A. glauca* and *A. mexicana*, induction was examined in two separate experiments. The first experiment (hereafter referred to as the “ontogeny experiment”), conducted June 15–July 07 2012, focused on the ontogenetic patterns of induction in *A. glauca* and tested whether induction of prickles, latex, leaf toughness differed between plants in the early (1–4 true leaves) and late (5–7 true leaves) juvenile ontogenetic stages, which corresponded to 2 and 4 weeks of age, respectively. The greatest changes in defense often occur during the transition from the seedling to juvenile and early juvenile stages of plants [1], [3]. In order to avoid confounding induction and ontogeny, separate individual plants were treated and assayed in the two ontogenetic stages. The second experiment (hereafter referred to as the “species comparison experiment”) focused on the comparison of *A. glauca* versus *A. mexicana* and included plants in the late juvenile stage (4–8 true leaves), at 5 weeks of age. The species comparison experiment began ten days after the ontogeny experiment was completed (July 17–August 01 2012).

In both experiments, induction was tested by randomly assigning plants to the following four treatment groups: (1) *Control* - in which plants did not receive any treatment; (2) *Damage* - in which plants were subjected to 50% mechanical defoliation by removing the distal half of all leaves with scissors; (3) *Jasmonic acid* - in which plants were sprayed with a jasmonic acid solution, 0.5 mM jasmonic acid solution with distilled water [50]; and (4) *Damage + Jasmonic acid Combination* - in which plants were subjected to 50% mechanical defoliation and sprayed with jasmonic acid. Mechanical damage and jasmonic acid were used as a test of induction because of the absence of native herbivores that could be used to damage *A. glauca*. Because of high specificity and coevolution in induced responses to native herbivores [33], substituting a novel invasive herbivore would not be appropriate. Artificial damage and jasmonic also provides greater generalizability, which is important when comparing species from such different communities and evolutionary histories. Replication per treatment group within genetic families was 2–3 plants. In the ontogeny experiment, a total of 19 *A. glauca* genetic families (15 from Maui and 4 from Hawai’i) were included, giving a total sample size of N = 339 plants. In the species comparison experiment, three genetic families per species (all from Hawai’i) were included, giving a total sample size of N = 67 plants.

Plants receiving the jasmonic acid treatment were temporarily transported downwind from the remaining plants and sprayed until the lower surfaces of all leaves (damaged and undamaged) were saturated. The upper leaf surface of both *A. glauca* and *A. mexicana* proved difficult to saturate due to the presence of thick epicuticular waxes. Plants that were not receiving jasmonic acid treatments were sprayed with water until dripping to control for possible effects of spraying on induction.

The final harvest occurred when each plant had developed at least two new leaves following the damage and jasmonic acid treatments (14 days after treatment for the ontogeny experiment and 16 days following treatment in the species comparison experiment). Because induction of physical defenses can only occur on newly developed leaves, we waited until the second leaf finished maturation in case the next leaf to develop after treatment applications had already begun to expand at the time of treatments. All traits were measured on this same leaf, including the amount of fresh latex exuded by the leaf upon excision (mg), prickle density on the upper (adaxial) and lower (abaxial) leaf surfaces quantified as the total number of prickles per leaf area (prickles/cm^2^), and specific leaf area (cm^2^/g) as a measure of leaf toughness [24].

The amount of latex was quantified by cutting the distal tip of the leaf and collecting the exuded latex onto a filter paper of known weight. Because Papaveraceae is characterized by having articulated laticifers that likely obstruct latex from fully draining out of the leaf tip [51], latex was also collected on the same filter paper after removing the leaf at the leaf base from the stem. The filter paper was then enclosed in a pre-weighed plastic vial and immediately weighed. The difference between the vial + filter paper with latex and the pre-weighed vial + filter paper represents fresh latex amount (mg). The excised leaf used to collect latex was then examined under 10x magnification to quantify prickle density. All prickles covering the adaxial and abaxial surfaces of the leaf were counted, excluding prickles found along leaf edges. A digital photo was then taken of the leaf, and leaf area was quantified using ImageJ [52]. The rest of the shoot was harvested, and all aboveground tissue was oven-dried at 60°C to constant weight, and dry biomass was measured to the nearest 0.01 mg.

### Statistical Analyses

Statistical analyses were conducted using SAS for Windows version 9.2 PROC MIXED (Cary, North Carolina). Residuals were examined for each variable, and data were log-transformed as needed to meet assumptions of normality and homoscedasticity. Type III sums of squares are reported for all analyses. Response variables analyzed in both experiments include: shoot biomass (g), specific leaf area (SLA, cm^2^/g), latex amount (mg), adaxial prickle density (number of prickles/cm^2^), abaxial prickle density (number of prickles/cm^2^).

In the ontogeny experiment, each variable was analyzed with a mixed-model ANCOVA that included the following factors: plant ontogenetic stage (early and late juvenile stages), island population (Maui, Hawai’i), genetic family nested within island, and treatment group (control, damage, jasmonic acid, damage + jasmonic acid). In addition to plant ontogenetic stage, plant size was accounted for by including the number of leaves at harvest time as a covariate. Genetic family was considered a random variable, and the significance of family and all interactions with family were tested by running the models with and without the random factor of interest, and then calculating the log-likelihood ratio statistics, which can be compared to a chi-square distribution with one degree of freedom (Littell *et al.,* 1996).

In the species comparison experiment, data were analyzed with mixed-model ANCOVAs that included the following factors: plant species (*A. glauca*, *A. mexicana*), genetic family nested within species, treatment group and leaf size as a covariate. Genetic family and interactions with genetic family were analyzed as random factors as in the ontogeny experiment.

In both experiments, significant effects of treatment group on defense traits would reveal induction of these traits. Tukey-adjusted least-square mean comparisons were used to identify patterns of induction. For example, a significant difference in prickle density between control plants and those in the damage group would reveal the induction of prickles by mechanical defoliation. A significant difference between damage vs. damage + jasmonic acid groups would reveal the effect of jasmonic acid in the induction of traits over and above that caused by mechanical damage. Significant variation among genetic families and between islands would indicate genetic variation, and significant interactions between genetic factors (family and island) and treatment would reveal genetic variation in induction.

## Results

### Ontogeny Experiment

**Figure 1:**
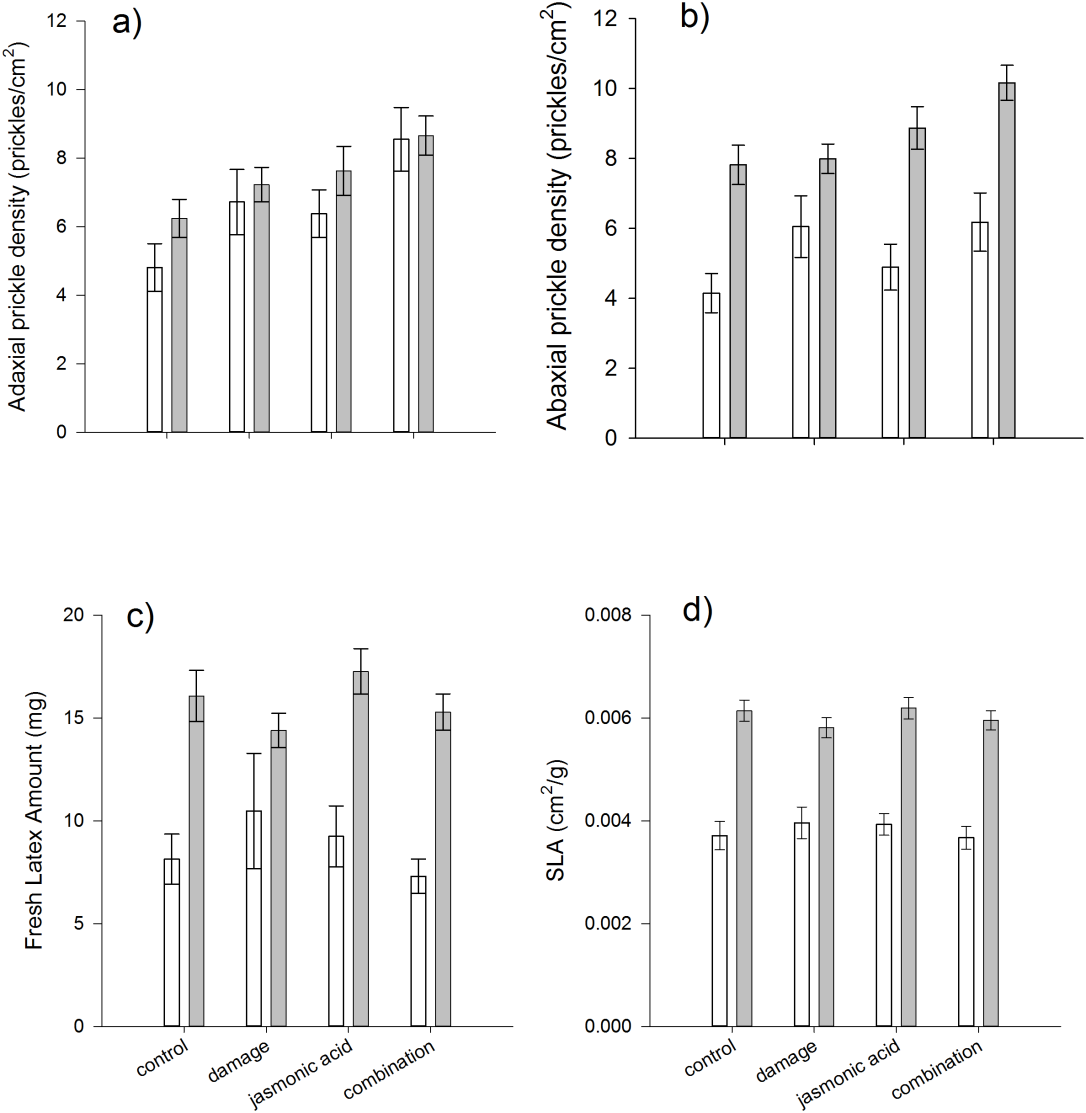
Effects of damage, jasmonic acid, and their combined application on (a) adaxial and (b) abaxial leaf surface prickle density (# prickles/cm^2^), (c) fresh leaf latex exudation (mg), and (d) specific leaf area (SLA, cm^2^/g). Young (clear bars) and old (grey bars) juvenile ontogenetic stages are contrasted, and bars are means ± 1 S.E.

Prickle densities in *A. glauca* were highly variable within and among all treatment groups, with adaxial prickle densities ranging from 0–29.3 prickles/cm^2^ and abaxial prickle densities ranging from 0–23.1 prickles/cm^2^. In contrast, latex and leaf toughness showed considerably less variation (Fig. 1).

Significant induction of prickles was detected on both leaf surfaces, and in both cases, it was the combined treatment of both mechanical damage and jasmonic acid application that had the highest prickle densities (Table 1, Fig. 1a, b). Adaxial prickle density was significantly higher in the combination treatment group than undamaged control plants (Tukey least-square mean comparison, *t* = 3.99, *P* = 0.0005), than plants receiving just mechanical damage (Tukey least-square mean comparison, *t* = 3.41, *P* = 0.0041) or just jasmonic acid (Tukey least-square mean comparison, *t* = 2.96, *P* = 0.0174). Abaxial prickle density was significantly higher in the combination treatment group than in the control group (Tukey least-square mean comparison, *t* = 3.81, *P* = 0.0010), but not significantly different from damage group (Tukey least-square mean comparison, *t* = –1.66, *P* = 0.3476), or jasmonic acid group (Tukey least-square mean comparison, *t* = –1.75, *P* = 0.2990). For both the adaxial and abaxial leaf surfaces, damage or jasmonic acid application alone did not significantly elevate prickle density above control levels (P>0.2990 for all pairwise comparisons), indicating the requirement for both mechanical damage and jasmonic acid to elicit the full induced response of *A. glauca*.

**Table 1:** Summary of mixed-model ANCOVA’s for the ontogeny experiment.

Variable	n	Isle (F)	Ontogeny (F)	Treatment (F)	Ontogeny×Treatment (F)	No. Leaves	Family (χ^2^)	Significant Interactions
SLA (cm^2^/g)	339	8.97**	88.46***	1.44	0.81	141.75***	4.5*	
Latex (mg)	338	10.05**	15.25**	1.82	3.15	63.81***	0	Isle×Ont×Treat (2.79*)Ont×Fam (4.0*)
Adaxial Density (prickles/cm^2^)	339	0.30	0.71	6.03**	5.74**	3.80^+^	45.6***	Isle×Ont×Treat (6.67**)
Abaxial Density (prickles/cm^2^)	339	0.40	19.0***	4.91**	0.82	19.36***	6.3**	

Fixed factors (F) were tested with F-test statistics, and random factors (χ^2^) were tested using log-likelihood ratio statistics compared to a chi-square distribution with one degree of freedom. The covariate is the number of leaves at harvest.

Significance is given as ***(P<0.0001), **(P<0.001), *(P<0.05), ^+^(P<0.07).

In contrast to prickles, neither latex nor leaf toughness (SLA) were induced by any of the treatments (Table 1, Fig. 1c, d).

Ontogeny influenced many of the traits measured, including the overall expression of prickles, latex, leaf toughness, and the induction of adaxial prickle density, revealed by a significant interaction between ontogeny and treatment (Table 1, Fig. 1). In general, it appears that older juvenile plants are better defended than younger juvenile plants, as evidenced by an increase in abaxial prickle density and latex exudation (Fig. 1). In contrast, leaf toughness decreased significantly with age (Fig. 1d). Although adaxial prickle density does not show a general change between ontogenetic stages, the magnitude of induction is higher in the young juvenile stage compared to the older juvenile stage (Fig. 1a), revealed as a significant ontogeny×treatment interaction (Table 1). In addition to the ontogeny factor, the significant relationship between the number of leaves as a covariate and all traits analyzed (Table 1) demonstrates the important role of plant size (and age/ontogeny) on the expression of defenses.

**Figure 2:**
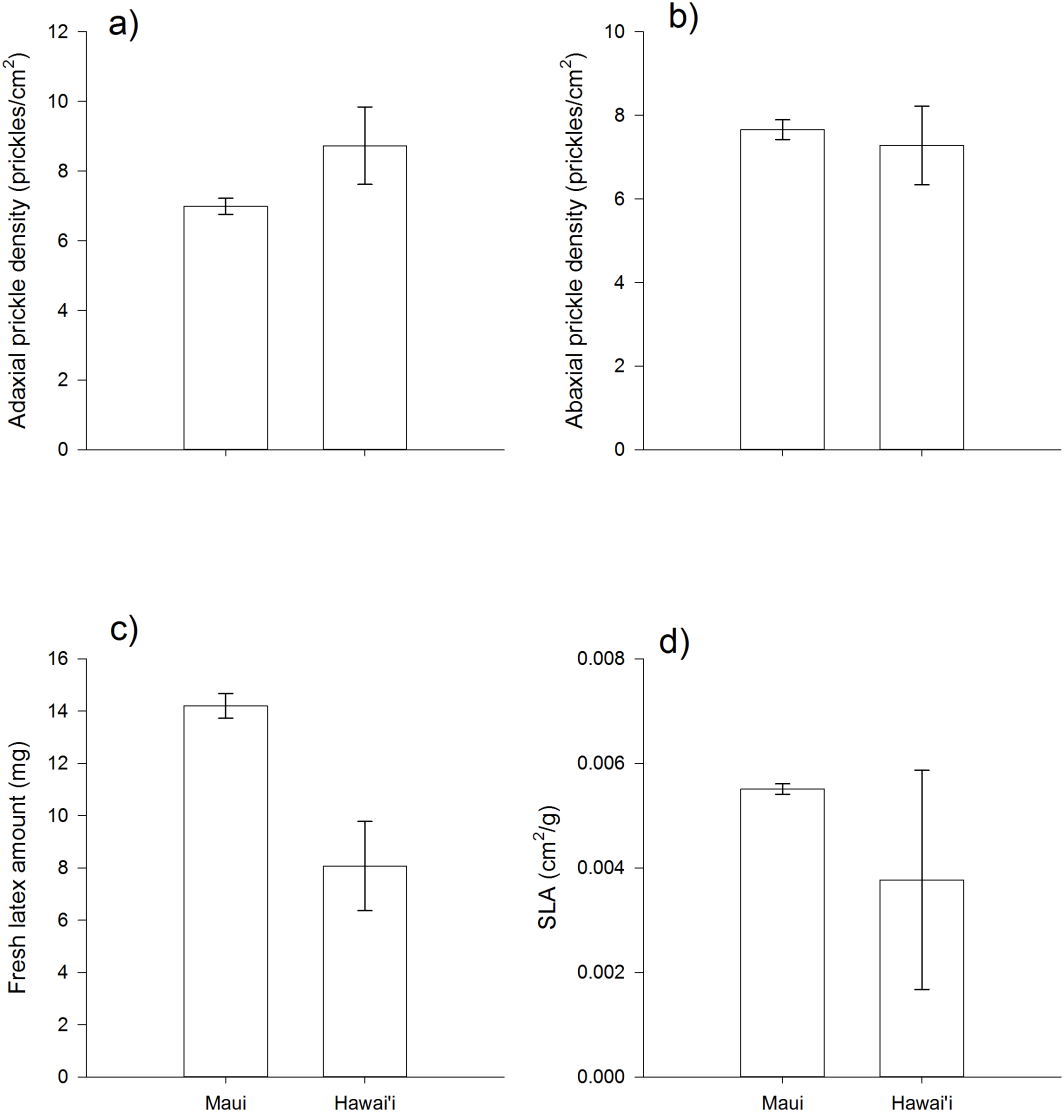
Population differences for the constitutive expression of (a) adaxial and (b) abaxial prickle densities, (c) latex amount, and (d) specific leaf area in *A. glauca* from the ontogeny experiment. Bars are means ± 1 S.E.

Significant variation among genetic families within populations was detected for leaf toughness (SLA) and prickle density on both leaf surfaces (Table 1). However, genetic variation was not detected for the induction of any of these traits (no significant family×treatment interactions). Genetic variation was also detected at the population scale, with significant differences between the two islands for leaf toughness and latex (Table 1, Fig. 2). Plants from Maui had leaves that were less tough but exuded more latex, than plants from Hawai’i (Fig. 2). Again, there is no evidence that induction differs between island populations, although there are weak three-way interactions between island×treatment×age for latex and adaxial prickles (Table 1) suggesting that ontogenetic patterns of induction of these two traits may differ between islands.

### Species Comparison Experiment

**Figure 3:**
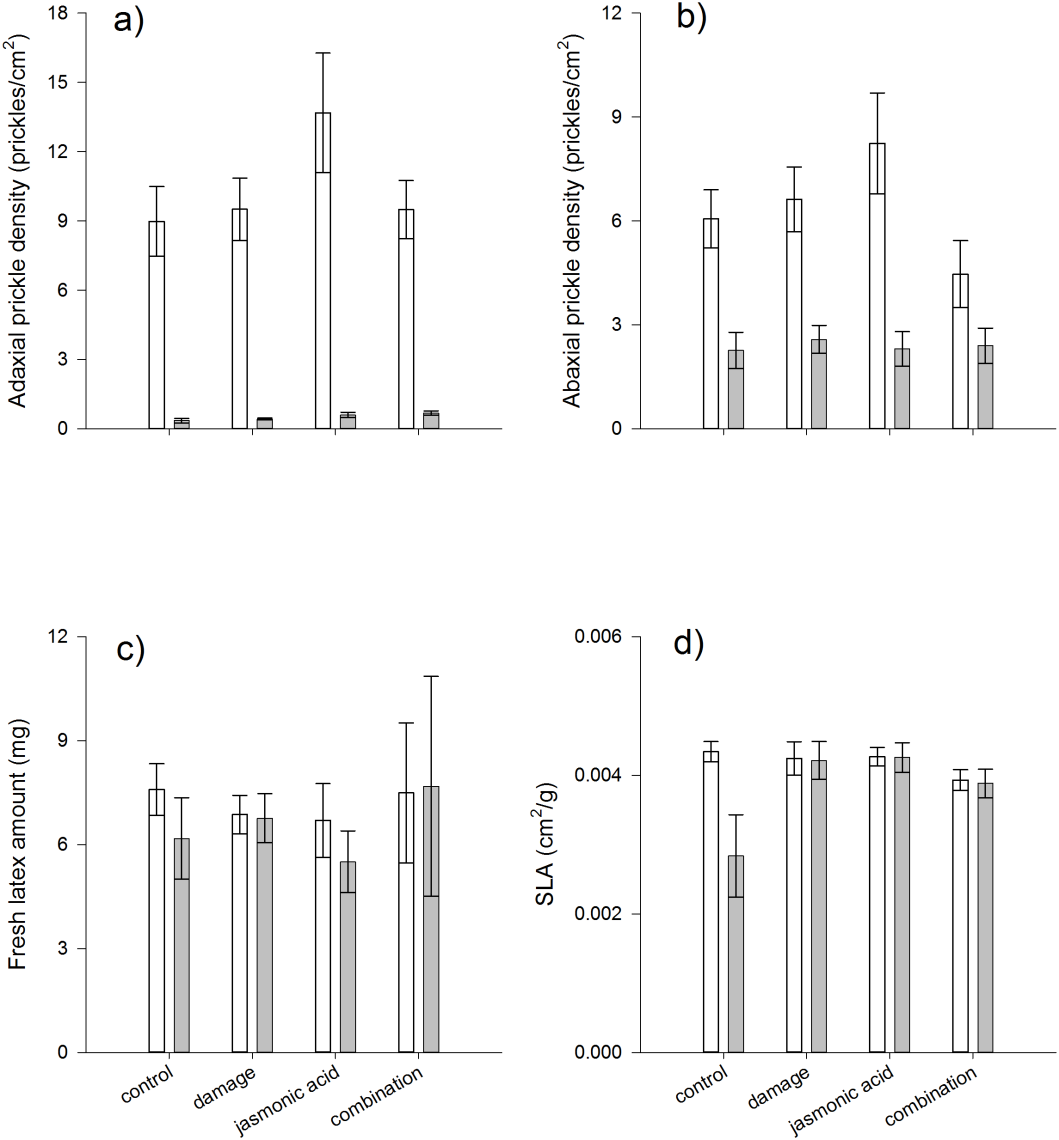
Effects of damage, jasmonic acid, and their combined application on (a) adaxial and (b) abaxial leaf surface prickle density (# prickles/cm^2^), (c) fresh leaf latex exudation (mg), and (d) specific leaf area (SLA, cm^2^/g). Patterns are presented for *Argemone glauca* (clear bars) and *A. mexicana* (grey bars), and bars are means ± 1 S.E.

Significant differences were detected between *A. glauca* and *A. mexicana* for leaf toughness, latex exudation, and prickle density on both leaf surfaces (Table 2). Species differences were particularly profound for prickles with *A. glauca* densities 20x and 2.7x higher than *A. mexicana* for adaxial and abaxial surfaces, respectively (Fig. 3a, b). *Argemone glauca* was also better defended in terms of latex and leaf toughness than *A. mexicana* (Fig. 3).

**Table 2:** Summary of results from mixed-model ANCOVA’s for the species comparison experiment.

Variable	n	Species (F)	Treatment (F)	Species×Treatment (F)	No. Leaves	Family (χ^2^)
SLA (cm^2^/g)	67	10.19**	1.10	3.65	12.21**	0
Latex (mg)	66	18.81***	2.37	0.39	36.37***	0
Adaxial Density (prickles/cm^2^)	67	83.68**	5.20**	1.15	6.18*	9.2**
Abaxial Density (prickles/cm^2^)	67	11.71*	1.76	1.99	0.68	7.9**

Fixed factors (F) were tested with F-test statistics, and random factors (χ^2^) were tested using log-likelihood ratio statistics compared to a chi-square distribution with one degree of freedom. The covariate is the number of leaves at the time of harvest.

Significance is given as ***(P<0.0001), **(P<0.001), *(P<0.05).

Both species demonstrated significant induction of prickles on the adaxial leaf surface (Table 2, Fig. 3a). However, in contrast to the ontogeny experiment, in this case, it was *A. glauca* plants subjected to jasmonic acid that showed the highest prickle densities (Fig. 3a, b). Neither latex nor leaf toughness was induced in either species (Table 2; Fig. 3c, d).

Prickle density (on both leaf surfaces), but not leaf toughness, latex, or the induction of any of these traits, varied significantly among maternal genetic families (Table 2).

Plant size (analyzed as the leaf number covariate) was again a significant contributing factor in the expression of all traits except abaxial prickle density (Table 2), confirming the important role of age/ontogeny in defense expression.

## Discussion

This study uncovers complex patterns of variation among three physical defense traits in prickly poppies. Most importantly, we revealed that plant ontogeny strongly influences the constitutive expression of all three defense traits, but only the inducibility of prickles. Moreover, the constitutive expression of latex increases across ontogeny while leaf toughness decreases. The ontogenetic decrease in leaf toughness was surprising considering the general tendency for young plant tissues to be less tough than older plant tissues [53]. Neither latex nor toughness were inducible at either ontogenetic stage tested.

Variable patterns of expression for prickles, latex and toughness point to their being unlinked genetically. This is in contrast to the covariance that is predicted when defense traits work synergistically as “syndromes” [54]. Previous research on *A. glauca* has similarly revealed that prickles, latex and damage tolerance differ in their patterns of variation with respect to island source, water availability, and mechanical damage, further emphasizing the independence of these physical defense traits in this species [23]. These patterns could indicate that the traits differ in function as well. For example, prickle density may function primarily in the ecophysiology of plants under high light, as evidenced by their induction in high light [45]. Considering the importance of trichomes for reflectance and water balance [55], [56], [57], this physiological function of prickles seems likely. Leaf toughness is also well known to provide physiological benefits under arid and high light environments [19], [58]. Considering their similar roles in defense and abiotic stress tolerance, we might expect that toughness and prickles would covary in prickly poppies, which was not the case. In contrast, prickles increased during ontogeny while toughness decreased. This could indicate that these traits are redundant and that plants undergo an ontogenetic switch from toughness to prickles in tolerance of abiotic stress.

Unlike prickles and leaf toughness, latex is likely to function solely in defense [59]. Latex increases with ontogeny, indicating that seedlings are less well defended than older juvenile plants. Because of the importance of seedling herbivory by non-native slugs and snails in Hawai’i [60], this pattern may make *A. glauca* vulnerable to these novel threats. Although latex amount did not increase in response to the induction treatments, we were unable to quantify the alkoids in the latex and so cannot rule out the possibility that latex induction is part of the *A. glauca* defense strategy.

In addition to ontogenetic variation, this study revealed considerable genetic variation in the constitutive patterns of expression in physical defense traits within and between islands in *A. glauca*. Plants from Maui showed significant prickle induction on both leaf surfaces while plants from Hawai’i induced prickles only on the adaxial leaf surface. These population differences could indicate geographic variability in herbivore selection pressure, or simply reflect variation due to genetic drift. Another possibility is that the sampling method introduced confounding variability which led to this result because seeds were collected from three sites on Hawai’i and from only one site on Maui. Future research quantifying herbivory levels on the two islands would shed light on these differences. In contrast to the constitutive expression of prickles, latex and leaf toughness, we detected no genetic variation in the induction of these traits, suggesting that their evolution by natural selection may be constrained. However, further sampling with additional genetic families is needed to confirm that genetic variation in induction is in fact absent from these populations and was not simply undetectable in the current study due to low sample sizes [61].

Our examination of the constitutive and induced expression of physical defenses in this sister species pair provides clear evidence against the idea that island plants have lower levels of defenses compared to continental plants [25], [26]. The endemic Hawaiian prickly poppy, *A. glauca*, has prickle densities nearly an order of magnitude higher than the continental Mexican poppy, *A. mexicana*, and the magnitude of prickle induction is also higher in *A. glauca*. Latex amount and leaf toughness are similar between these two species. Because *A. mexicana* has only been present in Hawai’i for about 50 years [35] and is likely to have experienced similar selection pressure as *A. glauca* by non-native herbivores during this time, the differences observed here are most likely due to their distinct evolutionary histories in continental vs. island communities. Future research examining levels of herbivory and fitness consequences for both species in the field would shed light on this. While previous studies have produced contradictory patterns, with examples of higher defenses in continental versus island plants [30], [62] and also examples of higher defenses in island plants [29], none of these studies have compared sister species or included prickles or latex in their surveys. Thus, we provide novel evidence that island plants may be better defended than predicted.

Clearly the evolution of plant defense on islands is more complex than what is considered by the island plant defense hypothesis. The most likely native herbivores to have selected for prickles, latex and leaf toughness in *A. glauca* are the now-extinct flightless ducks [40] and insects such as beetles. Currently, there are several new herbivores that may be selecting for defensive traits, such as non-native goats, deer and insects. Thus, while it is impossible to determine whether higher constitutive and induced expression of prickles in *A. glauca* compared to *A. mexicana* is due more to historical selection pressure by native herbivores or to current selection pressure by non-native herbivores, these data suggest that defense is well developed in this island endemic, providing strong evidence against the idea that selection pressure for plant defense is absent or weak on islands.

In closing, we provide a compelling example of ontogentic patterns of constitutive and induced expression of physical defense traits. Further evidence is needed to confirm that these responses to damage and hormonal application do in fact minimize damage from herbivores and increase the fitness of *A. glauca* and *A. mexicana*, and field studies would be particularly enlightening in this context. Nonetheless, this study captured the complexity of expression in defense traits in two well-defended plant species, and sheds light on how two sister species may become divergent in defense syndromes in island versus continental communities.
